# Root Morphology Was Improved in a Late-Stage Vigor Super Rice Cultivar

**DOI:** 10.1371/journal.pone.0142977

**Published:** 2015-11-13

**Authors:** Min Huang, Jiana Chen, Fangbo Cao, Ligeng Jiang, Yingbin Zou

**Affiliations:** 1 Collaborative Innovation Center of Grain and Oil Crops in South China, Hunan Agricultural University, Changsha, China; 2 Key Laboratory of Crop Cultivation and Farming System, Guangxi University, Nanning, China; National Taiwan University, TAIWAN

## Abstract

This study aimed to test the hypothesis that root morphology might be improved and consequently contributing to superior post-heading shoot growth and grain yield in late-stage vigor super rice. A pot experiment was carried out to compare yield attributes, shoot growth and physiological properties and root morphological traits between a late-stage vigor super rice cultivar (Y-liangyou 087) and an elite rice cultivar (Teyou 838). Grain yield and total shoot biomass were 7–9% higher in Y-liangyou 087 than in Teyou 838. Y-liangyou 087 had 60–64% higher post-heading shoot growth rate and biomass production than Teyou 838. Average relative chlorophyll concentration and net photosynthetic rate in flag leaves were 7–11% higher in Y-liangyou 087 than in Teyou 838 during heading to 25 days after heading. Y-liangyou 087 had 41% higher post-heading shoot N uptake but 17–25% lower root biomass and root-shoot ratio at heading and maturity than Teyou 838. Specific root length and length and surface area of fine roots were higher in Y-liangyou 087 than in Teyou 838 at heading and maturity by more than 15%. These results indicated that root-shoot relationships were well balanced during post-heading phase in the late-stage vigor super rice cultivar Y-liangyou 087 by improving root morphology including avoiding a too great root biomass and developing a large fine root system.

## Introduction

Rice is the staple food for a large segment of the world population [1], and global average rice yield must increase by more than 1.2% annually to meet the growing demand for food that will result from population growth and economic development [2]. To achieve this goal, great efforts should be made to breed new rice cultivars with higher yield potential to enhance average farm yields [3]. In 1996, China established a nationwide mega-project on the development of super rice based on the ideotype concept [4]. The ideotype was reflected in the following morphological traits: moderate tillering capacity (270–300 panicles m^–2^); heavy (5g panicle^–1^) and dropping panicles at maturity; plant height of at least 100 cm (from soil surface to unbent plant tip) and panicle height of 60 cm (from soil surface to the top pf panicles with panicles in natural position) at maturity; and long, erect, thick, narrow, and V-shaped top three leaves [3]. By 2015, 118 cultivars that met super rice criteria were released by provincial or national seed boards (http://www.ricedata.cn). It is reported that super rice cultivars have increased yield potential by more than 10% compared with ordinary rice cultivars [5, 6].

There have been several studies on the mechanism for the high yield in super rice [7–10]. From these studies it is clear that super rice cultivars can be classified into two types: (1) early-stage vigor type, which is characterized by large leaf area index and great shoot biomass production before heading [10]; and (2) late-stage vigor type, which has high leaf photosynthetic rate, slow leaf senescence and great shoot biomass production after heading [7–9]. Furthermore, it has been well documented that the superior shoot growth before heading in early-stage vigor super rice is related to improved root morphological and physiological traits including high root biomass, length density, oxidation activity and cytokinin content [10]. However, for late-stage vigor super rice, although it has been recognized that the superior shoot growth after heading is associated with improved root physiological traits including high cytokinin content and low abscisic acid content in roots [8], limited information is available on the relationships between shoot growth and root morphological traits.

Root biomass is regarded as one of the most important root morphological traits in rice [11]. However, too high levels of root biomass would not necessarily promote shoot growth. Samejima et al. [12, 13] observed that new plant type (NPT) rice lines had higher root biomass and root-shoot ratio but lower N uptake rate and relative shoot growth rate than the check cultivar IR72. Therefore, root biomass alone cannot adequately describe many root functions involved in root-shoot relationships, but that other root morphological traits, including root length, surface area and branching patterns, must be included [10, 11, 14]. The purpose of this study was to test the hypothesis that root morphology might be improved and consequently contributing to superior post-heading shoot growth and grain yield in late-stage vigor super rice. A pot experiment was conducted to compare yield attributes, shoot growth and physiological properties and root morphological traits between a late-stage vigor super rice cultivar and an elite rice cultivar.

## Materials and Methods

### Plant materials

Two rice cultivars, Y-liangyou 087 and Teyou 838, were used in this study. Y-liangyou 087 is an *indica* hybrid cultivar (Y58S × R087) released in 2010. This cultivar was approved as super rice by the Ministry of Agriculture of China in 2013. According to our preliminary studies in 2012 and 2013 (S1 File), Y-liangyou 087 is a late-stage vigor cultivar with high post-heading shoot growth rate and consequently high post-heading shoot biomass production, total biomass production and grain yield (S1 Table and S1 Fig). Teyou 838 is an *indica* hybrid cultivar (Longtepu-A × Fuhui 838) released in 2000. This cultivar is often used as a check cultivar because of its high grain yield and yield stability [15], and has been widely grown by rice farmers in China, especially in Guangxi Province, with a total area of approximately 600 × 10^3^ ha up to now (http://www.ricedata.cn).

### Experimental details

An outdoor pot experiment was carried out at the research farm of Guangxi University (22°51′ N, 108°17′ E, 78 m asl), Nanning, Guangxi Province, China in late-rice growing season in 2014. The site is located in a subtropical monsoon climate zone. Daily mean temperature and solar radiation during the rice-growing season were 25.4°C and 12.3 MJ m^–2^ d^–1^, respectively (Vantage Pro2 weather station, Davis Instruments Corp., Hayward, CA, USA). The soil used was collected from the upper 20 cm of a rice paddy field at the research farm. The soil was an Ultisol (USDA taxonomy) with pH 6.75, organic matter 32.3 g kg^–1^, NaOH hydrolysable N 120 mg kg^–1^, Olsen P 31.6 mg kg^–1^, and NH_4_OAc extractable K 126 mg kg^–1^. The soil was air-dried, sieved, and then filled in 40 plastic pots (length: 24 cm; width: 20 cm; height: 28 cm) with a depth of 20 cm.

Each cultivar was planted in 30 pots. Each pot was considered as one replication. Pre-germinated seeds were sown in seedling trays on 20 July. Uniformly-sized seedlings were selected at 20 days after sowing. Transplanting was done with one hill per pot and two seedlings per hill. Plants in each pot received 0.79 g N as urea, 0.26 g P_2_O_5_ as superphosphate and 0.86 g K_2_O as potassium chloride. N and K fertilizers were split-applied with 50% as basal, 30% at early-tillering and 20% at panicle initiation. P fertilizer was applied as basal. A floodwater depth of about 5 cm was maintained from transplanting until maturity. Insects and diseases were controlled by chemicals, and weeds were controlled by hand removal.

### Data collection

At heading, six pots with uniform plants were selected for each cultivar. Plants in the selected pots were uprooted and roots were detached from their nodal bases. The remaining roots in soil were carefully collected by handpicking. The roots from each pot were combined, washed and then scanned using a scanner (Epson Expression 1680 Scanner, Seiko Espon Corp. Tokyo, Japan). The scanning images were analyzed by a WinRHIZO root analyzer system (Regent Instruments Inc., Quebec, Canada) to determine root length, surface area and diameter. Roots were divided into two types according to the root diameter: fine (< 0.5 mm) and coarse roots (≥ 0.5 mm). Root biomass was determined after oven-drying at 70°C to a constant weight. Specific root length was calculated as the ratio of root length to root biomass. Tiller number was counted and shoots were separated into leaves, stems and panicles. Each organ was oven-dried at 70°C to a constant weight to determine pre-heading shoot biomass production (shoot dry weight at heading). Pre-heading shoot growth rate (pre-heading shoot biomass production/growth duration from transplanting to heading) and root-shoot ratio (root biomass/shoot biomass) were calculated. The dry shoot samples were ground for measuring N concentration by using a Skalar SAN Plus segmented flow analyzer (Skalar Inc., Breda, The Netherlands). Pre-heading shoot N uptake was calculated by multiplying pre-heading shoot biomass production by N concentration.

From heading to 25 days after heading, six pots with uniform plants were marked for each cultivar to determine relative chlorophyll concentration and net photosynthetic rate on the flag leaves of main stems at a 5-day interval. The relative chlorophyll concentration was determined by using a portable chlorophyll meter (SPAD-502, Konica Minolta, Osaka, Japan). The net photosynthetic rate was determined with a portable photosynthesis system (LI-6400, Li-Cor, Lincoln, NE, USA) at 09:00–10:30. It was measured at a light intensity of 1200 μmol m^–2^ s^–1^, a leaf temperature of 30°C, a constant CO_2_ concentration of 380 ± 5 μmol mol^–1^, and a relative humidity of 75 ± 5% in the sample chamber.

At maturity, roots and shoots were sampled from the marked plants. Root length, surface area, diameter and biomass and specific root length were determined according the methods described above. Tiller number was counted and shoot samples were separated into straw, rachis, and unfilled and filled spikelets. Each organ was oven-dried at 70°C to a constant weight to determine grain yield (adjusted to 14% moisture), total shoot biomass, harvest index, and total shoot N uptake. Root-shoot ratio, post-heading shoot biomass production (total shoot biomass—pre-heading shoot biomass production), post-heading shoot growth rate (post-heading shoot biomass production/growth duration from heading to maturity), and post heading shoot N uptake (total shoot N uptake—pre-heading shoot N uptake) were calculated.

### Statistical analysis

Data were subjected to analysis of variance (Statistix 8.0, Analytical Software, Tallahassee, FL, USA). Means of cultivars were compared based on the least significant difference (LSD) test at the 0.05 probability level.

## Results

### Yield attributes

Y-liangyou 087 produced 9% higher grain yield than Teyou 838 (Table 1). Total shoot biomass was 7% higher in Y-liangyou 087 than in Teyou 838. There was no significant difference in harvest index between the two cultivars.

**Table 1:** Yield attributes, shoot growth traits and shoot N uptake of rice cultivars Y-liangyou 087 and Teyou 838.

Parameter	Y-liangyou 087	Teyou 838
Grain yield (g pot^–1^)	75.7 (1.8)*	69.6 (0.9)
Shoot biomass production (g pot^–1^)		
Total	126 (3)*	117 (1)
Pre-heading	81.6 (2.3)*	90.5 (2.2)
Post-heading	44.1 (0.8)*	26.9 (1.3)
Harvest index (%)	52.1 (0.7)	51.3 (0.7)a
Growth duration (d)		
Pre-heading	68	63
Post-heading	39	38
Shoot growth rate (g pot^–1^ d^–1^)		
Pre-heading	1.20 (0.03)*	1.44 (0.04)
Post-heading	1.13 (0.02)*	0.71 (0.03)
Shoot N uptake (g pot^–1^)		
Pre-heading	0.82 (0.02)*	0.85 (0.02)
Post-heading	0.31 (0.02)*	0.22 (0.02)

Values in parentheses are SD (*n* = 6).

* indicates significant difference between the two cultivars according to LSD (0.05).

### Shoot growth and physiological properties

**Fig 1:**
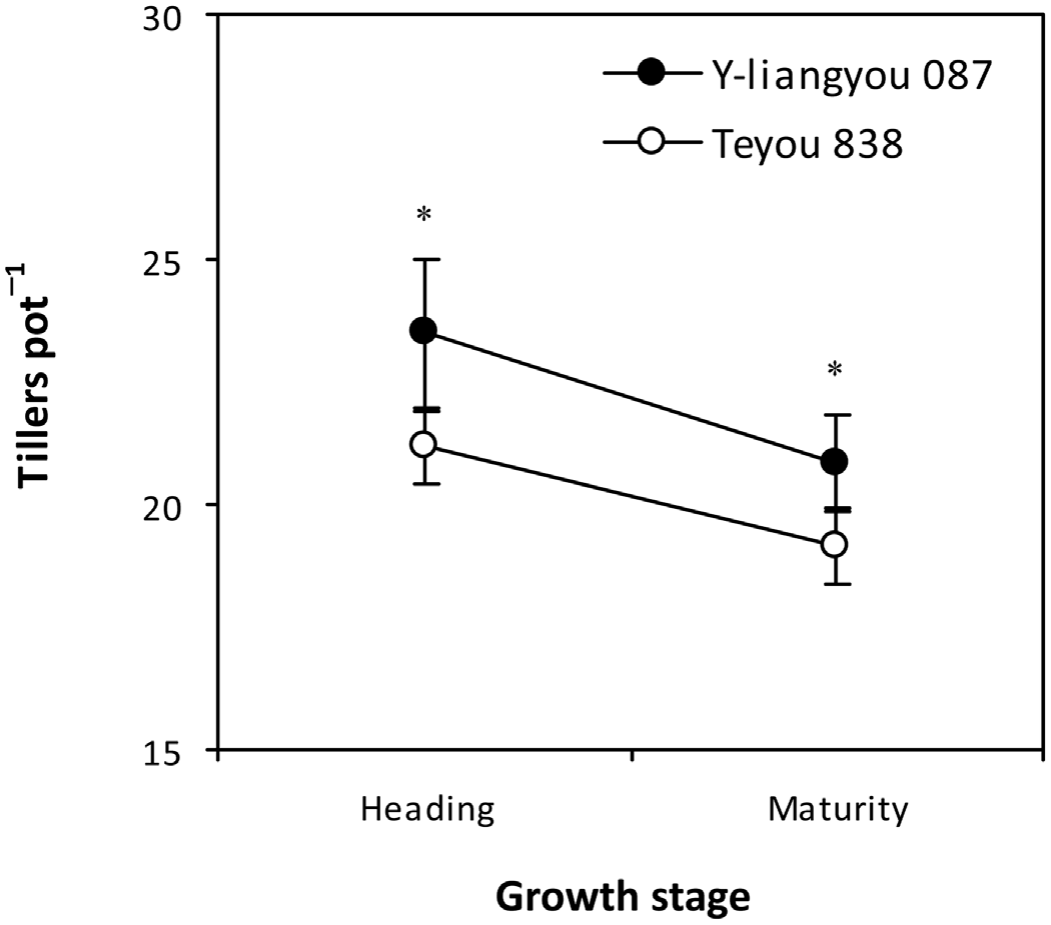
Tiller number at heading and maturity of rice cultivars Y-liangyou 087 and Teyou 838. Vertical bars represent SD (*n* = 6). * indicates significant difference between the two cultivars according to LSD (0.05).

Tiller number was 11% and 8% higher in Y-liangyou 087 than in Teyou 838 at heading and maturity, respectively (Fig 1). Pre-heading shoot biomass production was 10% lower in Y-liangyou 087 than in Teyou 838, whereas post-heading shoot biomass production was 64% higher in Y-liangyou 087 than in Teyou 838 (Table 1). Y-liangyou 087 had 5 d longer duration of pre-heading phase but similar duration of post-heading phase compared to Teyou 838. Pre-heading shoot growth rate was 17% lower in Y-liangyou 087 than in Teyou 838, while post-heading shoot growth rate was 60% higher in Y-liangyou 087 than in Teyou 838.

**Fig 2:**
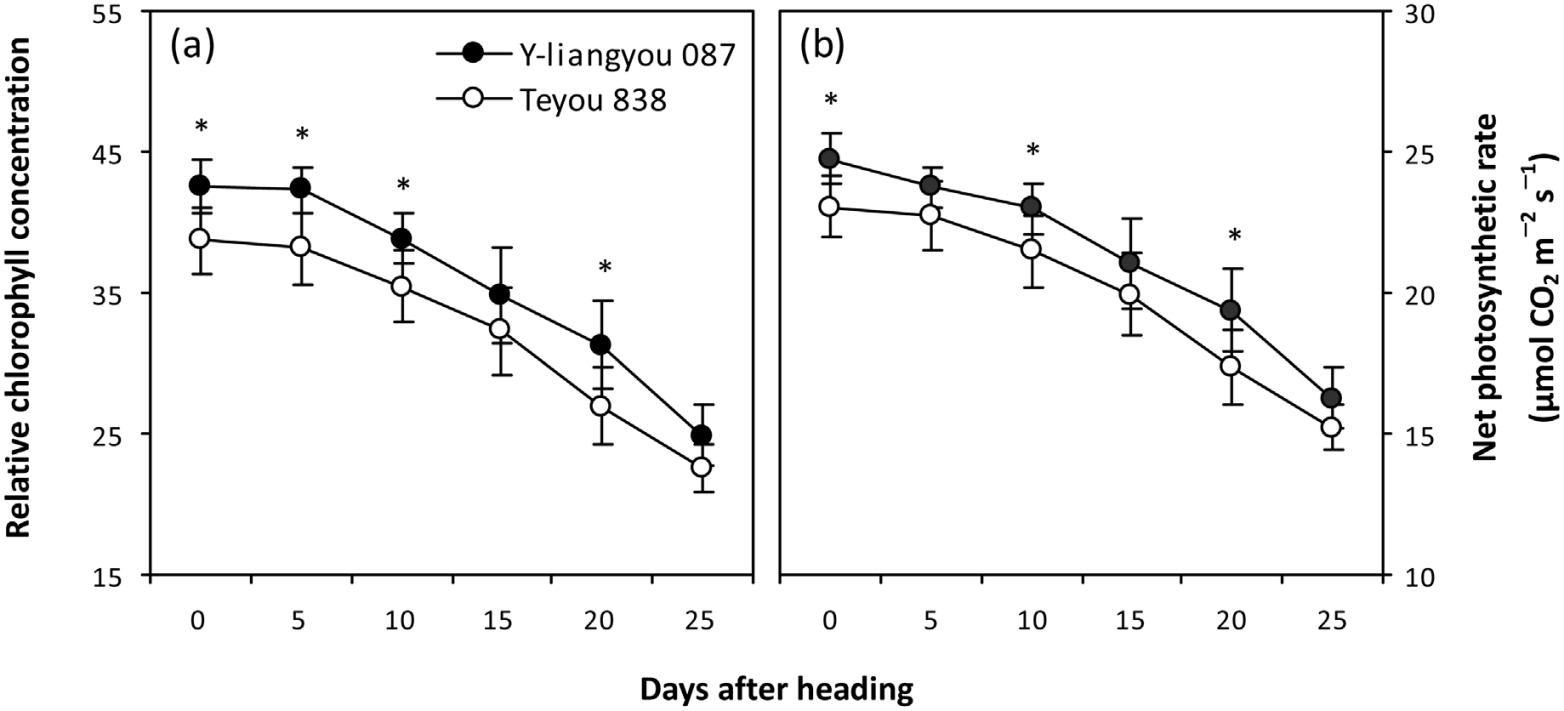
Relative chlorophyll concentration (a) and net photosynthetic rate (b) in flag leaves of rice cultivars Y-liangyou 087 and Teyou 838. Vertical bars represent SD (*n* = 6). * indicates significant difference between the two cultivars according to LSD (0.05).

Pre-heading shoot N uptake in Y-liangyou 087 was slightly (3%) but significantly lower than that in Teyou 838 (Table 1). Post-heading shoot N uptake was 41% higher in Y-liangyou 087 than in Teyou 838. From heading to 25 days after heading, Y-liangyou 087 generally had higher relative chlorophyll concentration and net photosynthetic rate than Teyou 838 (Fig 2a and 2b). Averaged across the period, relative chlorophyll concentration and net photosynthetic rate were 11% and 7% higher in Y-liangyou 087 than in Teyou 838, respectively.

### Root morphological traits

Y-liangyou 087 had 25% and 18% lower root biomass than Teyou 838 at heading and maturity, respectively (Table 2). Root length was not significantly differed between the two cultivars at heading, while it was 14% higher in Y-liangyou 087 than in Teyou 838 at maturity. Root surface area was 10% lower in Y-liangyou 087 than in Teyou 838 at heading, whereas the difference was not significant at maturity. Specific root length was 41% and 43% higher in Y-liangyou 087 than in Teyou 838 at heading and maturity, respectively. Root-shoot ratio in Y-liangyou 087 was lower than that in Teyou 838 by 17% at heading and by 24% at maturity.

**Table 2:** Root biomass, length and surface area, specific root length, and root-shoot ratio at heading and maturity of rice cultivars Y-liangyou 087 and Teyou 838.

Trait	Heading		Maturity	
	Y-liangyou 087	Teyou 838	Y-liangyou 087	Teyou 838
Root biomass (g pot^–1^)	13.8 (0.8)*	18.3 (1.2)	11.3 (0.8)*	13.9 (0.9)
Root length (m pot^–1^)	844 (52)	796 (79)	653 (48)*	571 (58)
Root surface area (m^–2^ pot^–1^)	1.06 (0.06)*	1.18 (0.12)	0.85 (0.06)	0.84 (0.09)
Specific root length (m g^–1^)	61.2 (3.8)*	43.4 (2.8)	57.8 (2.3)*	41.1 (2.1)
Root-shoot ratio	0.17 (0.01)*	0.20 (0.01)	0.09 (0.01)*	0.12 (0.01)

Values in parentheses are SD (*n* = 6).

* indicates significant difference between the two cultivars according to LSD (0.05).

**Fig 3:**
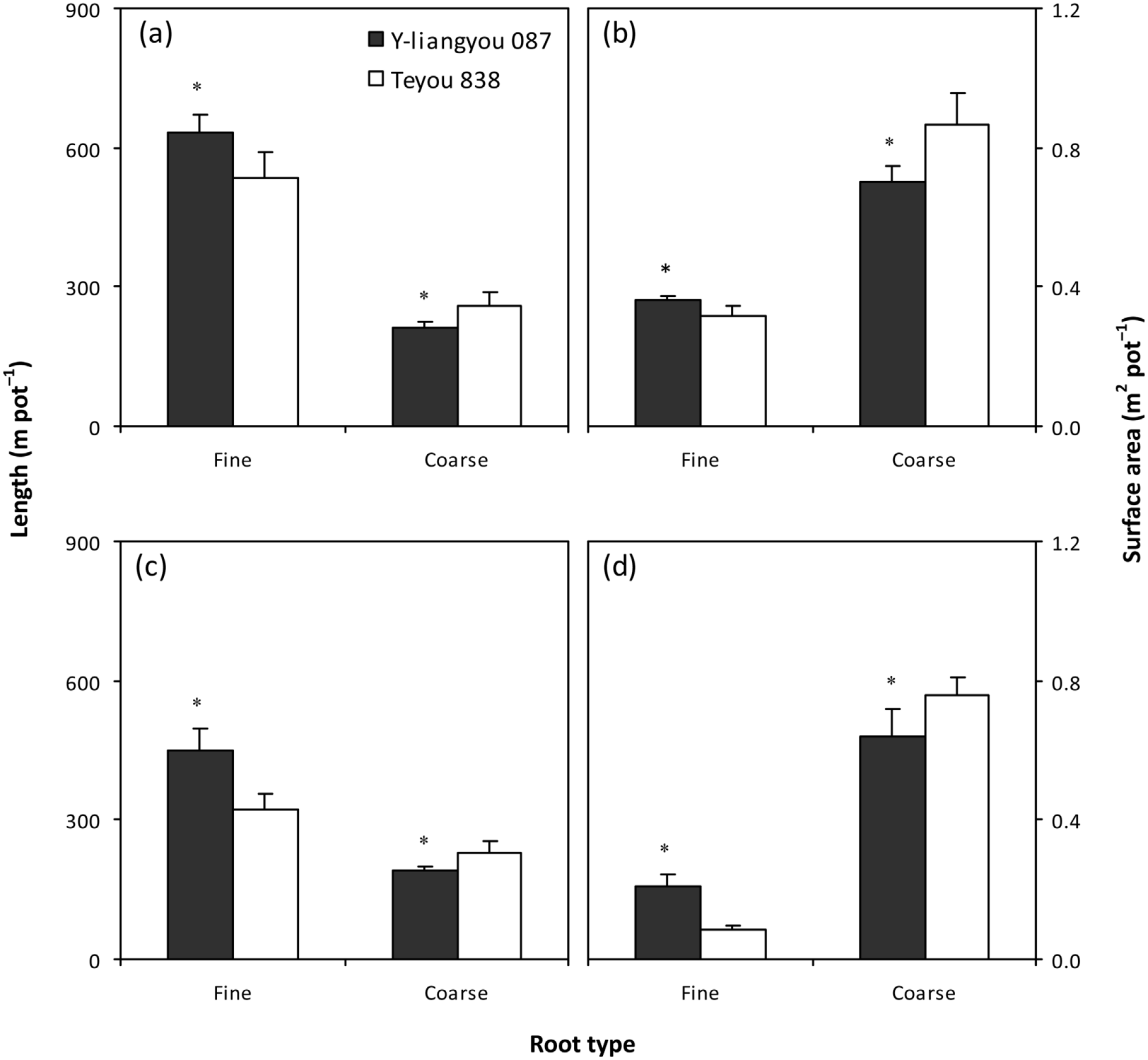
Length (a, c) and surface area (b, d) of fine and coarse roots at heading (a, b) and maturity (c, d) of rice cultivars Y-liangyou 087 and Teyou 838. Vertical bars represent SD (*n* = 6). * indicates significant difference between the two cultivars according to LSD (0.05).

Length of fine roots was 18% and 40% higher in Y-liangyou 087 than in Teyou 838 at heading and maturity, respectively (Fig 3a and 3c). On the contrary, length of coarse roots was lower in Y-liangyou 087 than in Teyou 838 by 19% at heading and by 17% at maturity. Y-liangyou 087 had 15% and more than 1.5 times higher surface area of fine roots but 19% and 16% lower surface area of coarse roots than Teyou 838 at heading and maturity, respectively (Fig 3b and 3d).

## Discussion

Consistent with the results of field experiments (S1 Table and S1 Fig), the present pot experiment showed that Y-liangyou 087 had higher post-heading shoot growth rate and thereby higher post-heading shoot biomass production, total biomass production and grain yield than Teyou 838. This again confirms that Y-liangyou 087 is a late-stage vigor super hybrid cultivar. High crop growth rate is the result of greater apparent canopy photosynthesis and/or less maintenance respiration [16]. In this study, the higher post-heading shoot growth rate in Y-liangyou 087 was partly attributed to improvement in leaf photosynthetic characteristics (higher relative chlorophyll concentration and net photosynthetic rate) during post-heading phase. This result is in agreement with that in the late-stage vigor super rice cultivar Xieyou 9308 [7, 17].

Prior to this study, there was limited information available on describing root morphological traits of late-stage vigor super rice. Our results showed that the late-stage vigor super rice cultivar Y-liangyou 087 had lower root biomass and root-shoot ratio than the check cultivar Teyou 838 at heading. This finding is not in agreement with that in the early-stage vigor super rice cultivars, which have high root biomass during the whole growing season [10]. In this regard, it is argued that the root is the organ for uptake of nutrients and water, but it also consumes assimilates produced by the shoot for root establishment and maintenance [11]. The energy used to produce root biomass is as 2-fold as that used to produce shoot biomass [18]. Based on this argument, the notion of root growth redundancy has been raised, namely, a too great root system could result in invalid consumption of energy and could be unfavorable to shoot growth [19–21]. In this study, the lower root biomass and root-shoot ratio in Y-liangyou 087 indicated that root growth redundancy was avoided in this cultivar. More interestingly, in the present study, the lower root biomass did not cause a lower post-heading shoot N uptake in Y-liangyou 087 than in Teyou 838. Instead, Y-liangyou 087 had about 40% higher post-heading shoot N uptake than Teyou 838. In fact, plant root system is not one organ but rather composed of two, and sometimes three, main types of root organs and not all the organs have the same functional abilities [22]. The root system of rice plants is comprised of coarse and fine roots, which correspond to seminal and nodal versus lateral roots [23]. Coarse roots serve functions of anchorage and typically establish overall root system architecture, controlling ultimate rooting depth, and the ability of plants to grown into compacted soil layers [24]. Fine roots are responsible for the bulk of nutrient and water acquisition [25]. In this study, Y-liangyou 087 had higher specific root length than Teyou 838 at both heading and maturity, indicating that lateral root formation was higher in Y-liangyou 087 than in Teyou 838 during post-heading. This was further supported by that length and surface area of fine roots (diameter less than 0.5 mm) were higher in Y-liangyou 087 than in Teyou 838 at heading and maturity. The observations indicated that the larger fine root system was an important reason for the higher post-heading shoot N uptake in Y-liangyou 087 than in Teyou 838. Moreover, we observed that length and surface area of fine roots were lower in Y-liangyou 087 than in Teyou 838 by 35–36% at mid-tillering and by 17–19% at panicle initiation (S2 Fig). This was partly responsible for the lower shoot N uptake and shoot biomass production during pre-heading phase in Y-liangyou 087 than in Teyou 838. These results indicated that fine root development might be delayed in Y-liangyou 087 as compared to Teyou 838. Root development is closely related with changes in tillering in rice [26]. In this study, tiller number was decreased from heading to maturity in both Y-liangyou 087 and Teyou 838 (Fig 1). This indicated that the large fine root system during post-heading phase in Y-liangyou 087 might be not attributed to further root system development. Consistently, length and surface area of roots including fine roots were decreased from heading to maturity in both the cultivars (Fig 3a–3d). More interestingly, it was found that the decreased magnitude of tiller number from heading to maturity was higher in Y-liangyou 087 (11%) than in Teyou 838 (9%) (Fig 1), whereas the decreased magnitudes of length and surface of fine roots from heading to maturity in Y-liangyou 087 (29–42%) were lower than those in Teyou 838 (40–74%) (Fig 3a–3d). This finding indicated that vitality of fine roots might be stronger in Y-liangyou 087 than in Teyou 838, and highlighting the need for greater fundamental understanding of the physiological processes governing large fine root system during post-heading phase in the late-stage vigor super rice cultivar Y-liangyou 087.

Taken together, it is clear that root-shoot relationships were well balanced during post-heading phase in the late-stage vigor super rice cultivar Y-liangyou 087 by improving root morphology including avoiding a too great root biomass and developing a large fine root system. This approach can be introduced to other breeding programs, such as IRRI’s NPT rice breeding program. It is reported that NPT lines had high root biomass and root-shoot ratio but low N uptake rate and relative shoot growth rate [12, 13], indicating that root growth redundancy was occurred in the NPT lines. This is to some extent why NPT lines have not increased the yield potential of rice [3]. We also suggest that root morphological traits should be considered as target components in ideotype rice breeding.

## Supporting Information

S1 File General details of field experiments. (PDF)

S1 Table Yield attributes under field conditions. (PDF)

S1 Fig Shoot growth characteristics under field conditions. (a), (c) and (e): pre-heading phase, (b), (d) and (f): post-heading phase. Data are means across two N rates. Vertical bars represent SD (*n* = 6). * indicates significant difference at the 0.05 probability level.
(TIF)

S2 Fig Fine root traits at mid-tillering and panicle initiation. MT: mid-tillering, PI: panicle initiation. Vertical bars represent SD (*n* = 6). * indicates significant difference at the 0.05 probability level.
(TIF)
