# Supplementary material for: Root Morphology Was Improved in a Late-Stage Vigor Super Rice Cultivar
Source: PLoS One. 2015 Nov 13;10(11):e0142977. doi: 10.1371/journal.pone.0142977 (PMC4643960; doi:10.1371/journal.pone.0142977)
Supplement: S1 File — (PDF) [file pone.0142977.s001.pdf]

S1 File. General details of field experiments.

Field experiments were conducted at the research farm of Guangxi University, Nanning, Guangxi Province, China (22°51′ N, 108°17′ E, 78 m asl) in late rice-growing seasons in 2012 and 2013. The site is located in a subtropical monsoon climate zone. The soil of the experimental field was an Ultisol (USDA taxonomy). The chemical properties of the upper 20 cm of the soil were pH 6.75, organic matter 32.3 g kg^{-1}, NaOH hydrolysable N 120 mg kg^{-1}, Olsen P 31.6 mg kg^{-1}, and NH_4OAc extractable K 126 mg kg^{-1}.

Treatments were arranged in a split-plot design with two N rates as main plots and two cultivars as subplots. The experiment was replicated three times and subplot size was 30 m^2. Two N rates were moderate and high N rates: 165 and 240 kg N kg^{-1}. For both N rates, urea was used as N fertilizer and was split-applied with 50% as basal (1 day before transplanting), 30% at early-tillering (7 days after transplanting) and 20% at panicle initiation. Two cultivars were Y-liangyou 087 and Teyou 838. Pre-germinated seeds were sown in a seedbed on 24 July. Twenty-day-old seedlings were transplanted with two seedlings per hill and a hill spacing of 20 cm × 20 cm. Superphosphate was used as P fertilizer with a rate of 54 kg P_2O_5 ha^{-1} and was applied as basal. Potassium chloride was used as K fertilizer with a rate of 180 kg K_2O ha^{-1} and was split-applied with 50% as basal, 30% at early-tillering and 20% at panicle initiation. The experimental field was kept flooded from transplanting until 7 days before maturity. Insects, diseases and weeds were intensively controlled by chemicals.

Plant shoots were sampled from a 0.48-m^2 area (12 hills) in each subplot at heading and maturity. Plant samples were separated into leaves, stems and panicles at heading, and into straw, rachis, and unfilled and filled spikelets at maturity. Each plant organ was oven-dried at 70 °C to a constant weight to determine pre-heading shoot biomass production (shoot dry weight at heading) and total shoot biomass production (shoot dry weight at maturity). Post-heading shoot biomass production (total shoot biomass – pre-heading shoot biomass), pre-heading shoot growth rate (pre-heading shoot biomass production/growth duration from transplanting to heading), post-heading shoot growth rate (post-heading shoot biomass production/growth duration from heading to maturity), and harvest index (100 × filled spikelet weight/total shoot weight) were calculated. Grain yield was determined from a 5-m^2 area in each subplot and adjusted to the standard moisture content of 0.14 g H_2O g^{-1}. Data were subjected to analysis of variance (Statistix 8.0, Analytical Software, Tallahassee, FL, USA). Means of cultivars were compared based on the least significant difference test at the 0.05 probability level.
