# Supplementary material for: Root Morphology Was Improved in a Late-Stage Vigor Super Rice Cultivar
Source: PLoS One. 2015 Nov 13;10(11):e0142977. doi: 10.1371/journal.pone.0142977 (PMC4643960; doi:10.1371/journal.pone.0142977)
Supplement: S1 Table — (PDF) [file pone.0142977.s002.pdf]

S1 Table. Yield attributes under field conditions.

N level	Cultivar	Total shoot biomass ($g\ m^{-2}$)		Harvest index (%)		Grain yield ($t\ ha^{-1}$)	
		**2012**	**2013**	**2012**	**2013**	**2012**	**2013**
Moderate N	Y-liangyou 087	1616	1550	52.3	51.1	9.04	8.42
	Teyou 838	1396	1391	51.6	50.7	8.17	8.02
	Mean	1506	1471	52.0	50.9	8.61	8.22
High N	Y-liangyou 087	1664	1625	49.8	48.6	9.64	8.96
	Teyou 838	1532	1466	51.0	49.3	8.32	7.83
	Mean	1598	1546	50.4	49.0	8.98	8.40
Analysis of variance	N rate	6.86^{*}	8.97^{*}	5.76^{ns}	6.90^{*}	1.14^{ns}	0.56^{ns}
	Cultivar	25.2^{**}	40.2^{**}	0.15^{ns}	0.02^{ns}	9.74^{*}	11.0^{*}
	N rate × Cultivar	1.55^{ns}	0.00^{ns}	2.33^{ns}	0.48^{ns}	0.42^{ns}	2.53^{ns}

*, ** *F*-value significant at the 0.05 and 0.01 probability level, respectively. ^{ns} *F*-value not significant at the 0.05 probability level.
