# Supplementary figures and images for: Root Morphology Was Improved in a Late-Stage Vigor Super Rice Cultivar
Source: PLoS One. 2015 Nov 13;10(11):e0142977. doi: 10.1371/journal.pone.0142977 (PMC4643960; doi:10.1371/journal.pone.0142977)

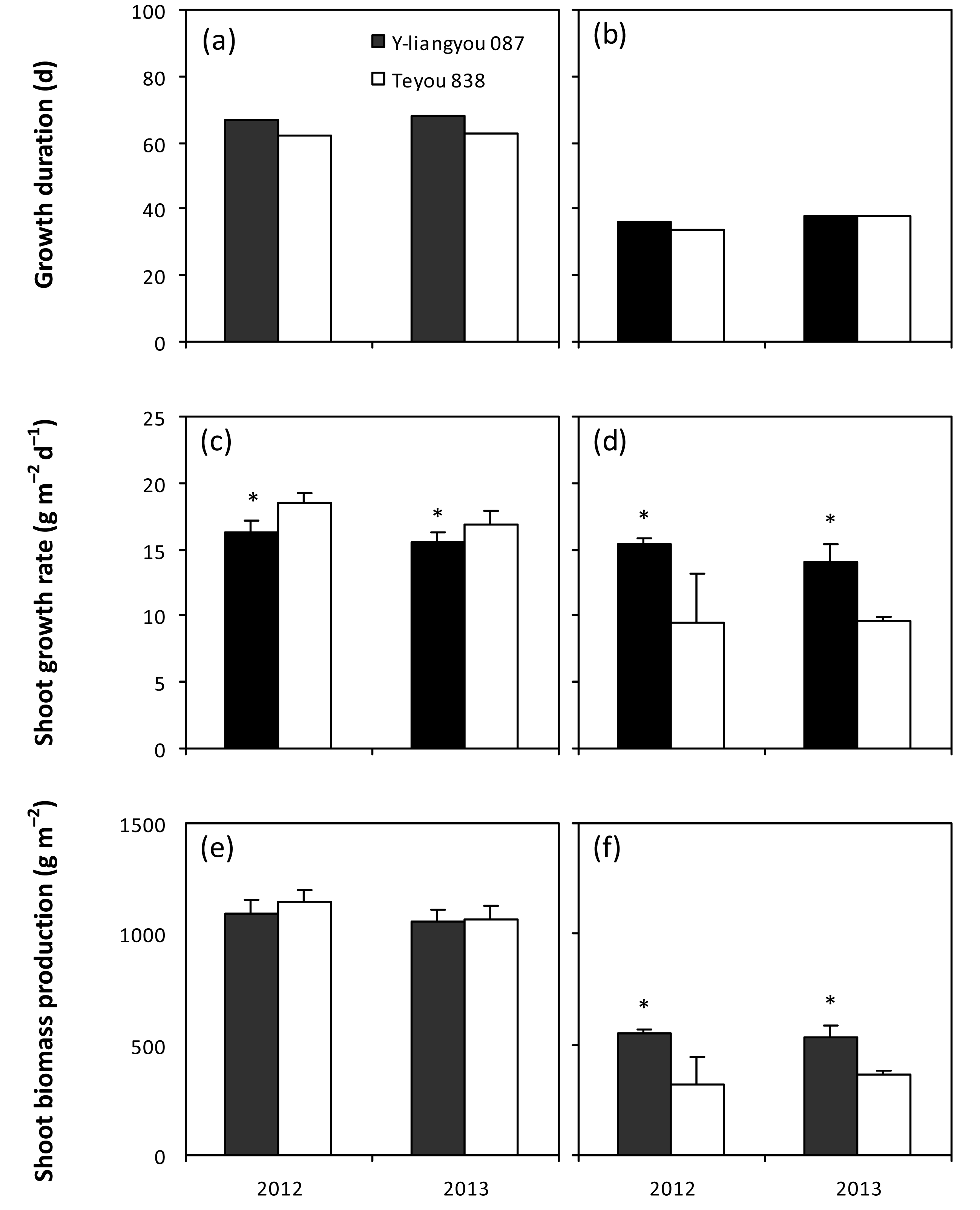

Supplement: S1 Fig — (a), (c) and (e): pre-heading phase, (b), (d) and (f): post-heading phase. Data are means across two N rates. Vertical bars represent SD (n = 6). * indicates significant difference at the 0.05 probability level. (TIF) [file pone.0142977.s003.tif]

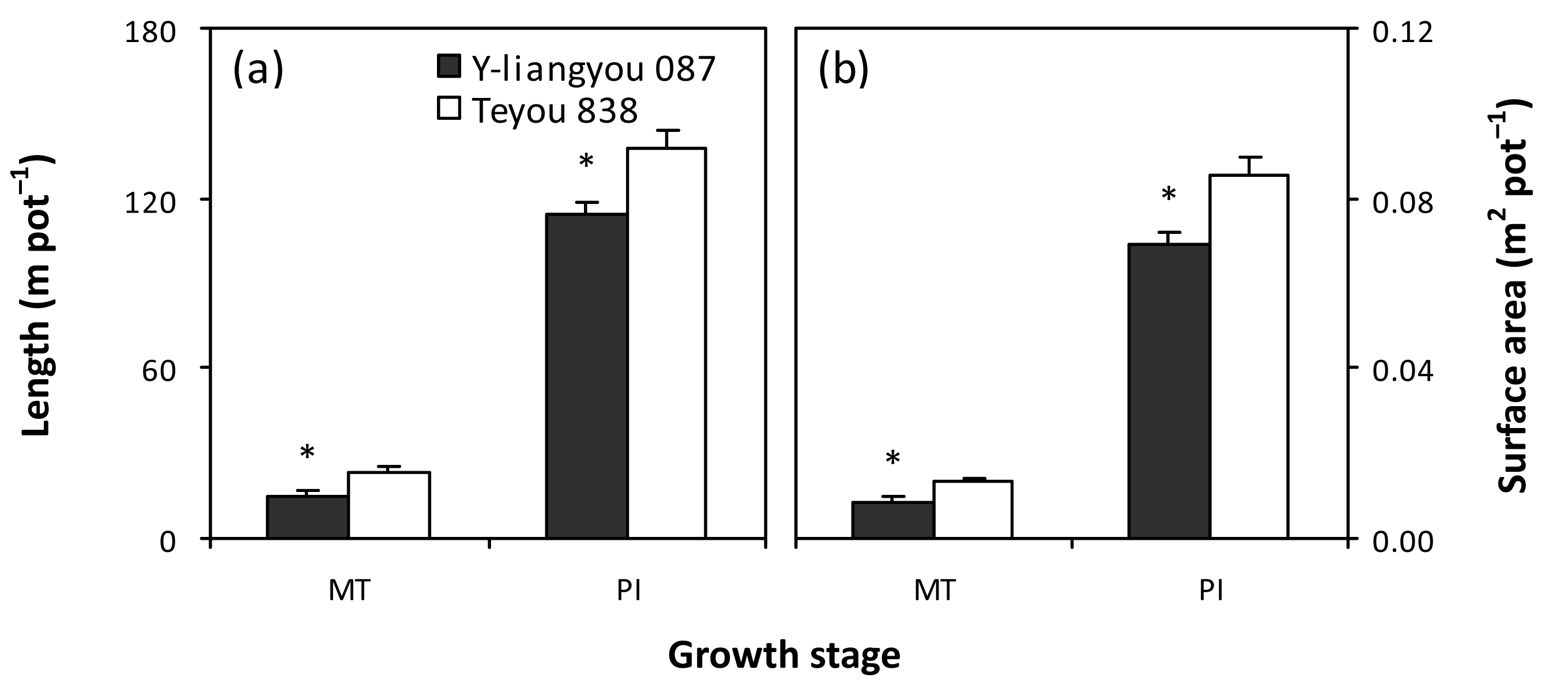

Supplement: S2 Fig — MT: mid-tillering, PI: panicle initiation. Vertical bars represent SD (n = 6). * indicates significant difference at the 0.05 probability level. (TIF) [file pone.0142977.s004.tif]
